# Looking forward to 2025: EUPHA is here for U!

**DOI:** 10.1093/eurpub/ckae216

**Published:** 2025-01-17

**Authors:** Tit Albreht, Charlotte Marchandise, Hans Henri P Kluge, Tine Rikke Jorgensen, Danilo Lo Fo Wong, Ketevan Kandelaki, Ilmo Keskimäki, Floris Barnhoorn

**Affiliations:** President of EUPHA; Executive Director of EUPHA; WHO Regional Director for Europe; Antimicrobial Resistance Programme, WHO EURO; Regional Advisor, Antimicrobial Resistance Programme, WHO EURO; Technical Officer, Antimicrobial Resistance Programme, WHO EURO; Chair of the 18th EPH Conference; Deputy-Director EPH Conference

Returning from Lisbon after the inspiring European Public Health EPH Conference that gathered more than 3000 participants, we are filled with gratitude and excitement for what lies ahead. It was a scientific conference; it was also a celebration of our collective dedication to advancing public health across Europe and beyond. The energy, collaboration, and innovative spirit we witnessed have set the stage for an ambitious and transformative journey toward 2025. 

This journey is also about creativity and storytelling. Inspired by the success of the song “*Where Health Can Thrive*” performed in Lisbon, our new anthem, we aim to continue embracing culture as a powerful tool to promote public health. Capturing the stories, struggles, and victories of public health will help make our mission more relatable and engaging for broader audiences.

## Embracing our new strategic pillars: evidence, equity, engagement, and empowerment

As we step into the future, EUPHA’s Governing Board has approved a new strategy guided by four pillars: “Evidence, Equity, Engagement, and Empowerment.” These pillars are the foundation of our mission to bridge science and policy, empower communities, and foster a resilient public health workforce.

- *Evidence*: Advance science-based policy and practice by fostering high-quality, actionable research. We aim to empower researchers and public health professionals, supporting them in translating and applying evidence to shape impactful policies, strategies, and communications.
- *Empowerment*: Build capacity and continuous education by equipping public health professionals, National Public Health Associations (NPHAs), and communities with the knowledge, tools, education, and autonomy to drive meaningful change. Partnerships will amplify NPHAs’ voices, ensuring their influence on European and global public health strategies.
- *Equity*: Champion inclusiveness and reduce inequalities by addressing the specific health needs of Europe’s diverse communities. We are committed to promoting culturally sensitive care that leaves no one behind and encouraging active participation to foster equity in all dimensions of health.
- *Engagement*: Foster co-creation and cross-sectoral partnerships by including community voices at every step of public health progress. By building bridges across sectors and countries, we tackle complex health challenges—such as pandemics, climate change, and digital health—with a focus on shared decision-making and resilience.

## A bold vision for the future

As we look to 2025, our vision is clear: to create a public health ecosystem in Europe that is “evidence-based, innovative, inclusive, and resilient.” Public health is increasingly at risk—from the rise of populism and disinformation to the devastating effects of war, conflict, and the erosion of trust in science. These challenges threaten the very foundations of equitable and evidence-based health systems. Now, more than ever, we must act boldly and collaboratively to protect and advance public health for all.

Together, with the support of our dedicated members, partners, and the broader public health community, we will turn our bold ideas into reality.

As Paulo Freire wisely said, “*As long as I fight, I am moved by hope; and if I fight with hope, then I can wait*.” With hope and determination, “EUPHA is here for U”—not just to discuss solutions, but to make them happen.

## Vintage innovation—bacteriophages as a valuable addition to the toolbox to fight antimicrobial resistance?

KlugeHans Henri P
WHO Regional Director for Europe
JorgensenTine Rikke
Antimicrobial Resistance Programme, WHO EURO
WongDanilo Lo Fo
Regional Advisor, Antimicrobial Resistance Programme, WHO EURO
KandelakiKetevan
Technical Officer, Antimicrobial Resistance Programme, WHO EURO

**EJPH**

**“WHO contribution”**

Antimicrobial resistance (AMR) is one of the top global health threats of our time, potentially reversing significant medical breakthroughs of the past century. Over the next 25 years, 39 million people are expected to die from AMR globally if no action is taken (https://www.sciencedirect.com/science/article/pii/S0140673624018671?via%3Dihub). In the WHO European Region (European Union Member States plus Iceland, Liechtenstein, and Norway), AMR is responsible for 133 000 deaths each year already (https://pubmed.ncbi.nlm.nih.gov/36244350/), with a projection that it could cost €11.7 billion due to healthcare costs and productivity losses annually in the EU/EEA countries alone (https://search.oecd.org/health/Fighting-Antimicrobial-Resistance-in-EU-and-EEA-countries-2023.pdf).

Acknowledging the scale of the AMR crisis and the need for action, last year the 53 Member States of the WHO European Region adopted the AMR Roadmap 2023–30 (https://iris.who.int/bitstream/handle/10665/372503/73wd07e-AMR-Roadmap-230574.pdf?sequence=5). Member States agreed that innovation and research will be key enablers to achieve the goal of preventing and controlling AMR.

One promising approach—which has been widely neglected so far in Western Europe—is the use of bacteriophages (phages).

Phages are viruses that selectively kill bacteria. What makes them so interesting is the fact that they can also kill bacteria that no longer respond to antibiotics treatment.

The history and experience in phage therapy is particularly extensive in the Eastern part of the WHO European Region. Many countries in Eastern Europe have used phage therapy for over 100 years, typically in combination with antibiotics as well as prophylaxis for larger populations, for example for soldiers during Soviet times.

### All too good to be true?

This all might sound too good to be true. The question “Why are phages not used widely already?” quickly comes up.

As so often, the answer is: it is not that simple.

Unlike antibiotics, phages are not approved as medicines (biological medicinal products) in most countries. So far, they are primarily used on compassionate grounds in Western Europe, in life-threatening situations, when all other treatments have been exhausted. Before phages could be available more widely for therapeutic use in humans, further evidence is needed from clinical studies to inform decision-making and robust regulatory frameworks.

This, backed by earmarked strategic funding, could go a long way to support the development and application of phage therapy.

### Not only relevant for human health

The AMR crisis requires a One Health approach considering the nexus of human, animal, and environmental health. The fact that phages also hold great potential for animal and environmental health makes them even more interesting. In veterinary medicine, agriculture and aquaculture, phages are already used more widely. They are used to treat infections in livestock and companion animals. As an example, phages have already been used to treat diarrhea caused by *Escherichia coli* in pigs and Metritis in cows (https://www.ncbi.nlm.nih.gov/pmc/articles/PMC8150778/).

In agriculture, phages are applied to control bacterial diseases in crops such as potatoes, while in aquaculture, they are used against infections in farmed fish. All these methods help to reduce the overall use of antibiotics, one of the major drivers of the AMR crisis (see for example: Application of bacteriophages and endolysins in aquaculture as a biocontrol measure—ScienceDirect; genomic epidemiology and Phage-based Prevention of salmon associated Pasteurella (GP3) (fhf.no); Bacteriophages as a Strategy to Protect Potato Tubers against Dickeya dianthicola and Pectobacterium carotovorum Soft Rot—PMC (nih.gov)).

### Utilizing the full potential of bacteriophages

In close collaboration with the Global AMR Research and Development Hub, the WHO Regional Office for Europe (WHO/Europe) is currently investigating the potential practical applications and evidence-based supporting the use of phages.

As part of this broader effort, WHO/Europe and the Global AMR R&D Hub are facilitating leading experts’ guidance on ways of accelerating evidence development on bacteriophages within the One Health framework (webinar—towards building the evidence for broader use of bacteriophages from an AMR One Health perspective (who.int)).

### Bacteriophages alone will not be the solution

Tapping into the potential of bacteriophages is a promising way forward for the long-term fight against drug-resistant bacteria infections.

This is not to say that phages alone will be the solution to the AMR crisis. Antimicrobial stewardship to educate and support health care professionals to follow evidence-based guidelines for prescribing and administering antimicrobials, coupled with improved knowledge and awareness among the general public on appropriate use of antimicrobials are some of the other tools that remain crucial in our efforts to control AMR.

Given the extent and acceleration of the AMR crisis, however, now is the best time to explore phages as a critical addition to the toolbox, complementing antibiotics for stronger efficacy.

## Investing for sustainable health and well-being

KeskimäkiIlmo
Chair of the 18th EPH Conference
BarnhoornFloris
Deputy-Director EPH Conference

18th European Public Health Conference

11–14 November 2025

Finlandia Hall, Helsinki, Finland

### Welcome to Helsinki, capital of the happiest country in the world

After the successful conference in Lisbon, we will meet again in person for the 18th European Public Health Conference in Helsinki, Finland, from 11 to 14 November. Helsinki will host the 18th EPH Conference in 2025, inviting you to reconnect with colleagues from around the globe to discuss key public health issues, explore the city, and have fun.

Known for its efficiency and safety, Helsinki is ranked the 11th best city for congresses worldwide. Additionally, Finland has been named the world’s happiest country for the seventh consecutive year in the World Happiness Report. This ranking is attributed to strong social support networks, social trust, honest governance, safe environments, and healthy lives. These topics will also be featured in the conference program.

### Main theme: investing for sustainable health and well-being

The 18th EPH Conference highlights that improving population health and well-being is an investment, not an expense. This investment benefits society and fosters a sustainable economy. Health and well-being are intricately linked with sustainable development: investing in health supports social, economic, and environmental sustainability, while a healthy planet with inclusive growth and fair societies enhances individual and community well-being. Addressing the determinants of health and reducing inequities are crucial for achieving population health and are essential for sustainable development.

### Subthemes: Economy of well-being and public health

- The economy of well-being emphasizes the interconnectedness of the economy, social security, healthcare, planetary boundaries, and overall welfare, focusing on long-term sustainability. It prioritizes people’s well-being in policy-making. Current economic systems, which contribute to environmental crises, social inequalities, and rising non-communicable diseases, are failing to generate true well-being. Addressing these issues requires shifting from a growth-centric model to one that measures and promotes well-being through integrated and consistent policies.

### Cultural diversity and inclusivity in sustainable health policy and practice

- Cultural diversity and inclusivity are crucial for sustainable health policy and practice. Recognizing diverse cultural perspectives in healthcare improves outcomes, fosters innovation, and strengthens resilience. Inclusive practices enhance patient satisfaction, trust, and equity by respecting cultural beliefs and involving marginalized communities in decision-making.

### Public health in the era of misinformation and disinformation

- Misinformation and disinformation pose increasing challenges to public health, spreading rapidly and often amplified by malicious bots and social media algorithms. These falsehoods can erode trust in healthcare systems, fuel vaccine hesitancy, and promote risky behaviors. Vulnerable populations are particularly at risk, which can deepen health disparities and create barriers to effective care. For public health professionals, it is crucial to explore strategies to combat these threats, ensuring accurate information prevails.

### Transforming economies for planetary health and well-being

- Human health and the environment are intertwined. Promoting co-beneficial solutions requires a multifaceted approach, including eco-friendly and health-promoting practices, innovative sustainable technologies, policy advocacy, and community engagement.

### Tackling political and commercial determinants of health through policy and governance

- This theme emphasizes the importance of collaboration between governments, organizations, and communities to create a sustainable health environment. It calls for transparency, accountability, and the involvement of diverse stakeholders in policy-making to foster resilient and inclusive health systems. Addressing the impact of political decisions and commercial interests on public health is crucial. Effective policies and governance can mitigate negative health outcomes. Prioritizing public well-being over profit through regulations, and ensuring political frameworks support health equity and access to healthcare for all, are essential.

### Investing in health workforce to build sustainable health systems

- The health workforce plays a critical role in achieving sustainable and resilient health systems. Adequate funding and resources are essential not only for training healthcare professionals but also for retaining them, ensuring continuous professional development, and maintaining high standards of care. A well-supported workforce, with a focus on their well-being, is key to system efficiency and positive health outcomes.
- Helsinki, the capital of Finland, combines urban culture, serene nature, a stunning archipelago, and vibrant urban areas. The city’s compact size makes navigation easy, allowing extensive exploration during your stay. Despite potentially gloomy November weather, we encourage you to experience Finnish hospitality and our efficient way of making things happen.
- We look forward to welcoming you to Helsinki!

